# Effectiveness of a Program Combining Strengthening, Stretching, and Aerobic Training Exercises in a Standing versus a Sitting Position in Overweight Subjects with Knee Osteoarthritis: A Randomized Controlled Trial

**DOI:** 10.3390/jcm9124113

**Published:** 2020-12-20

**Authors:** Betsy Denisse Perez-Huerta, Belén Díaz-Pulido, Daniel Pecos-Martin, David Beckwee, Enrique Lluch-Girbes, Ruben Fernandez-Matias, María José Bolaños Rubio, Tomas Gallego-Izquierdo

**Affiliations:** 1 Centro de Rehabilitación y Educación Especial Puebla SNDIF, Carretera a la Calera s/n Col. Lomas de San Miguel C.P., Puebla 72573, Mexico; betsy.pe.hu@gmail.com; 2 Department of Physiotherapy, University of Alcalá, 28871 Madrid, Spain; belen.diazp@uah.es (B.D.-P.); tomas.gallego@uah.es (T.G.-I.); 3 Physiotherapy and Pain Research Group, University of Alcalá, 28871 Madrid, Spain; 4 Rehabilitation Research Group, Department of Physiotherapy, Human Physiology and Anatomy, Vrije Universiteit Brussel, 1090 Brussels, Belgium; David.Beckwee@vub.be; 5 Department of Physiotherapy, University of Valencia, 46010 Valencia, Spain; enrique.lluch@uv.es; 6 Department of Physiotherapy, Human Physiology and Anatomy, Vrije Universiteit Brussel, 1090 Brussels, Belgium; 7 Pain in Motion Research Group, International Research Group, Vrije Universiteit Brussel, 1050 Brussels, Belgium; 8 Research Institute of Physiotherapy and Pain, University of Alcalá, 28871 Madrid, Spain; ruben.fernanmat@gmail.com; 9 Research Unit, Hospital Universitario Fundación Alcorcón, Alcorcón, 28922 Madrid, Spain; 10 Physiotherapist, Rehabilitation Center Rehavitalis, 28020 Madrid, Spain; marijo_yy888@hotmail.com

**Keywords:** exercise therapy, knee osteoarthritis, overweight, middle aged, position

## Abstract

There is an increasing incidence, prevalence, and burden of knee osteoarthritis due to a global increase in obesity and an aging population. The aim of the present study was to compare the effectiveness of the addition of aerobic exercises performed in an unloaded or loaded position to a conventional exercise program in overweight subjects with knee osteoarthritis. Twenty-four subjects were randomly allocated to receive 36 sessions of 30-min duration of either sitting aerobic exercises (experimental group) or standing aerobic exercises (control group). Pain intensity, knee disability, and quality-of-life data were collected at baseline and at 12, 24, and 36 sessions. Generalized linear mixed models (GLMMs) were constructed for the analysis of the differences. Significant differences were found in the experimental group for self-reported pain and knee pain and disability at 24 and 36 sessions (*p* < 0.05). Significant between-group differences were observed in change in self-reported knee pain and disability and quality of life from baseline to 24th- and 36th-session measurements in favor of the experimental group. Adherence to treatment was higher in the experimental group. Adding aerobic exercises in an unloaded position to a conventional exercise program produced superior effects over time for self-reported knee pain, knee pain and disability and quality of life compared to loaded aerobic exercises in overweight subjects with knee osteoarthritis.

## 1. Introduction

Osteoarthritis (OA) is one of the most common musculoskeletal conditions in our society [1,2], which affects over 80% of the population beyond the age of 55 [3]. Together with the hip, the knee is the most commonly affected joint with both sharing a predominantly load-bearing function [4]. There is an increasing incidence, prevalence, and burden of knee osteoarthritis due to the global increase in obesity and an aging population [5]. In addition to pain, knee OA causes stiffness, limited mobility, swelling, joint instability, and muscle weakness, all of which can lead to impaired physical function and reduced quality of life [6]. Factors such as age, overweight, and obesity play an important role in the development and aggravation of knee OA as they may accelerate cartilage degradation and then facilitate the development of OA [6,7].

Most clinical guidelines recommend exercise and weight loss for the management of knee OA in overweight patients [8,9,10]. Regarding exercise, the most effective programs include strengthening aquatic and/or aerobic exercises with all of them showing positive effects on patient-reported pain, disability, and quality of life in people with knee OA [11,12,13,14]. While the magnitude of exercise benefits may be considered small to moderate, these effects are comparable to those obtained with simple analgesics and oral nonsteroidal anti-inflammatory drugs, but exercise has much fewer side effects [15,16].

Being overweight may contribute to knee OA by increasing the load on the joint, effective methods for implementing exercises while reducing joint overload have emerged including aquatic and cycling exercise training. While these unloading strategies may be interesting to those patients who cannot tolerate therapeutic load training due to pain, they are often not considered the treatment of choice for knee OA. Performing some of the recommended exercises for the management of knee OA (e.g., aerobic exercise) in unloading positions (e.g., sitting position) might help to reduce some of the drawbacks of load training exercises, such as an increase of pain or the inability of many patients to remaining long-term in a loaded knee position. However, to our knowledge, studies directly comparing the clinical benefit of aerobic exercises performed in unloaded versus loaded conditions are lacking. In addition, evidence about the effects of aerobic training for overweight subjects with knee OA either when applied alone or in combination with other exercise modalities is scarce [12].

Adherence to exercise is associated with better treatment outcomes in people with knee OA [16,17]. Indeed, poor adherence is considered the most likely explanation for the declining impact of the benefits of exercise over time in people with knee OA [18], so focusing on how exercise behavior can be stimulated and maintained in the long term in this population has become a research priority [18].

The aim of this study was to evaluate the effects on knee pain, disability, and quality of life of adding unloaded aerobic exercises (seated) versus aerobic exercises in a loading condition (standing) to a conventional strengthening and stretching program in overweight subjects with knee OA. It was hypothesized that participants receiving the aerobic exercises in an unloading position would show significantly greater clinical improvements and adherence to treatment as compared to those performing aerobic exercises in a loading position.

## 2. Methods

### 2.1. Study Design

A two-arm, parallel-group, assessor-blinded, randomized-controlled trial conforming to CONSORT guidelines [19] was performed between June 2015 and February 2016 at the Specialization Center for Diagnosis and Treatment of Azuqueca de Henares, Spain. The study was approved by the Ethics Research and Experimentation Committee of the University of Alcala (CEIM/HU/2015/29) and conducted in accordance with the Declaration of Helsinki. The study was registered at Australian New Zealand Clinical Trials Registry (trial registration ACTRN12616000506493).

### 2.2. Participants

Subjects with knee OA aged between 50 and 80 years old were invited to participate in the study. They were recruited from the primary care rehabilitation area, Specialization Center for Diagnosis and Treatment of Azuqueca de Henares, Spain.

Individuals were included if they had symptomatic knee OA according to the American College of Rheumatology classification criteria [20]. These criteria were found to be 89% sensitive and 88% specific for diagnosing knee OA [20]. In addition, individuals were included if they had a body mass index (BMI) > 25 kg/m^2^.

Subjects were excluded if they were unable to walk or exercise without functional aids, had previous knee surgery, or were diagnosed of an unstable medical or psychiatric condition or advanced osteoporosis. Subjects were informed about the procedures and gave written informed consent prior to participation.

### 2.3. Sample Size

The required sample size was calculated using G*Power 3.0.18 software (Universität Düsseldorf, Düsseldorf, Germany) based on self-reported knee pain on the Visual Analogue Scale (VAS) as the primary outcome measure. The effect size (ES) for the self-reported knee pain was considered as ES = 0.25, and the correlation between repeated measurements was assumed at 0.5. Assuming four measurements (pre, post 12 sessions (post12), post 24 sessions (post24), and post 36 sessions (post36)) in the two treatment groups, the correction of sphericity was determined at 1. With a statistical power of 0.80 and an alpha level of 0.05, a total sample size of 24 patients was estimated. Considering a possible loss to follow-up of up to 10%, a total of 26 patients were recruited (13 per group).

### 2.4. Procedure

Demographic information including age, sex, weight, age, and BMI was first collected by self-report. Participants additionally completed a Visual Analogue Scale (VAS) to quantify their pain intensity.

They then completed the following self-administration questionnaires in a standardized order: The Western Ontario and McMaster Universities Arthritis Index (WOMAC) and the Short Form-12 Health Survey questionnaire (SF-12). Finally, treatment adherence from all participants was noted with a self-administered record.

A physiotherapist, specifically trained in all aspects of the assessment, was responsible for all the measurements. This assessor was blinded to the questionnaire data and treatment allocation.

### 2.5. Outcome Measurements

The primary outcome measure of this study was patient reported level of pain rated on the VAS. Secondary outcomes were results from the WOMAC and SF-12 questionnaires and treatment adherence. Every outcome was measured at baseline and after completing 12, 24 and 36 sessions of treatment. Treatment adherence was measured asking patients how many sessions of that prescribed they completed. 

#### 2.5.1. Self-Reported Knee Pain

Participants were asked to indicate the intensity of their pain in the last week on the VAS, which consists of a continuous 100-mm-long horizontal line where one end (left) corresponds to no pain and the other end (right) to worst possible pain. Test–retest reliability and internal consistency of the VAS have been demonstrated in people with knee OA [21]. In addition, VAS scores have shown good correlation with scores obtained in other pain descriptive scales [22]. A 20 mm change is required for the result of the VAS to be clinically meaningful in subjects with knee OA [21].

#### 2.5.2. Knee Pain and Disability

The Spanish version of the Western Ontario and McMaster Universities Osteoarthritis Index (WOMAC) was used [23]. The WOMAC comprises five items for pain (range 0–20), two for stiffness (range 0–8), and 17 for functional limitation (range 0–68). Total WOMAC score (range 0–96) and scores from the pain, stiffness, and functional subscales were considered in this study. Higher scores on the WOMAC indicate worse pain, stiffness, and functional limitations.

The test–retest reliability, internal consistency, convergent validity and responsiveness of the Spanish version of the WOMAC have been demonstrated in people with hip and knee OA [23]. A 7.9-point change is required for the result of the WOMAC to be clinically meaningful [21].

#### 2.5.3. Quality of Life

The Spanish version of the Short Form Survey (SF-12) was used to evaluate quality of life [24]. It is comprised of 12 items divided into two subscales: physical health composite (SF12PCS) (range 0–100) and mental health composite (SF12MCS) (range 0–100). The total SF-12 score (range 0–100) [25] and scores from the SF12PCS and SF12MCS subscales were considered in this study. Lower scores on the SF-12 indicate lower level of health.

Psychometric properties of the SF-12 were previously reported showing good internal consistency and reliability [26,27]. In addition, the criterion-related validity of SF-12 is satisfactory when comparing its results with those obtained in the SF-36 showing correlations ranging from 0.94 to 0.96 (physical summary) and from 0.94 to 0.97 (mental summary) [26,27]. The minimal detectable change score for the SF12PCS and SF12MCS in subjects with knee OA is 2.7 points and 3.5 points, respectively [27].

### 2.6. Interventions

An equal number of participants were randomly allocated using opaque sealed envelopes to receive either a program combining strengthening and stretching together with aerobic training exercises performed in an unloaded sitting position (experimental treatment) or the same strengthening and stretching program but with aerobic exercises applied in a loaded standing position (control treatment).

Both programs involved a total of 36 treatment sessions (3 sessions per week) [12] during the twelve weeks of the study. The first 12 sessions were performed in the clinic, in group, and supervised by a physiotherapist with a master’s degree in Musculoskeletal Physiotherapy, whereas the rest of the sessions (24 sessions) were performed by participants at home individually, using the exercises previously taught in the clinic and with digital versatile disc (DVD) guidance. Each session comprised 30 min of aerobic training performed in an unloaded (sitting—experimental group) or loaded (standing—control group) position depending on the allocated treatment, followed by 30 min of a series of strengthening and stretching exercises. Both groups received the same strengthening and flexibility exercises so the only difference between groups was the aerobic exercise program. In order to improve adherence to treatment, researchers contacted each patient by phone every 15 days to encourage them to continue with the treatment program.

All interventions were applied by a physiotherapist experienced in providing all treatment procedures. This physiotherapist was blinded to results of measurements and questionnaires used as outcome measures. Participants were instructed to continue with their current medications during the treatment period.

#### 2.6.1. Experimental Treatment

The experimental group started each session with 30 min of aerobic exercise in an unloaded sitting position structured as follows: 5 min of warm-up, 20 min of aerobic training, and 5 min of cooling-down. Warm-up included three exercises of 1 min duration with a 1 min interval between exercises (Supplementary Material S1). The same exercises, but in the reverse order, were used for cooling-down. Aerobic training comprised four series of five exercises of 1 min duration (Supplementary Material S2). Subjects were asked to rate their self-perceived exertion in a 10-point verbal BORG CR10 scale, ranging from 0 (rest) to 10 (maximal exertion). They were asked to maintain a 4/10 (moderate) self-perceived exertion during aerobic exercise.

After aerobic training, all participants performed lower limb isometric strengthening exercises, as these have been suggested to produce a hypoalgesic effect [28] and to be beneficial in these kind of patients [29], addressing the quadriceps, hamstrings, triceps surae, adductors, and abductors muscles. The exercise load prescribed for each exercise was one set of ten repetitions holding 10 s of contraction and 2 s rest between contractions using medium-resistance elastic bands and vinyl balls (Supplementary Material S3). Finally, stretching exercises targeting the above-mentioned muscles were performed in each lower limb for a total of one set of ten 10 s repetitions, with 2 s rest between repetitions using resistance bands if necessary (Supplementary Material S4). The duration of the strengthening and stretching exercises was 30 min.

#### 2.6.2. Control Treatment

The control group started each session with 30 min of aerobic exercise in a loaded standing position structured identically with regard to time as per the experimental group. However, the 5 min of warm-up and cooling-down included alternating 3 min of gentle walking with 2 min rest. Aerobic training in this group consisted of 20 min of moderate-intensity exercise (brisk walking). Moderate intensity was defined asking the subjects to rate their self-perceived exertion in a 10-point verbal BORG scale, ranging from 0 (rest) to 10 (maximal exertion), and being 4/10 a moderate exertion. Afterward, the same series of strengthening and stretching exercises as per the experimental group were performed for 30 min.

### 2.7. Statistical Analysis

Data were analyzed with the SPSS v22 software package for windows (SPSS Inc. version 22.0, IBM, Chicago, IL, USA). All statistical tests were performed considering a 95% confidence interval (CI) with an α level < 0.05. Per protocol analyses were conducted. Data normality was evaluated with the Shapiro–Wilk test. All data were normally distributed but for exercise adherence. For the descriptive analysis of continuous variables, the mean and standard deviation (SD) were reported. For nominal variables, the absolute frequencies were calculated. Homogeneity analysis was performed with Student’s *t*-test for continuous variables and with Pearson’s chi-square test or Fisher’s exact test for nominal variables.

For the differences between groups, generalized linear mixed models (GLMM) were constructed, with time (baseline, post-treatment, 2 months, and 3 months) as a within-subjects factor and group (experimental, control) as a between-subjects factor. Sphericity was evaluated with the Mauchly test, and the Greenhouse–Geisser correction was used when the sphericity assumption was not fulfilled. Partial eta squared (η_p_^2^) was used as an estimator of the effect size of the main effects and interactions of the GLMM. Post hoc comparisons were conducted with Student’s *t*-test with the Bonferroni correction. Effect sizes of post hoc comparisons were estimated with Cohen’s d, with the formula d = 2t/√g.

Differences in exercise adherence were analyzed with Mann–Whitney U-test.

## 3. Results

**Figure 1:**
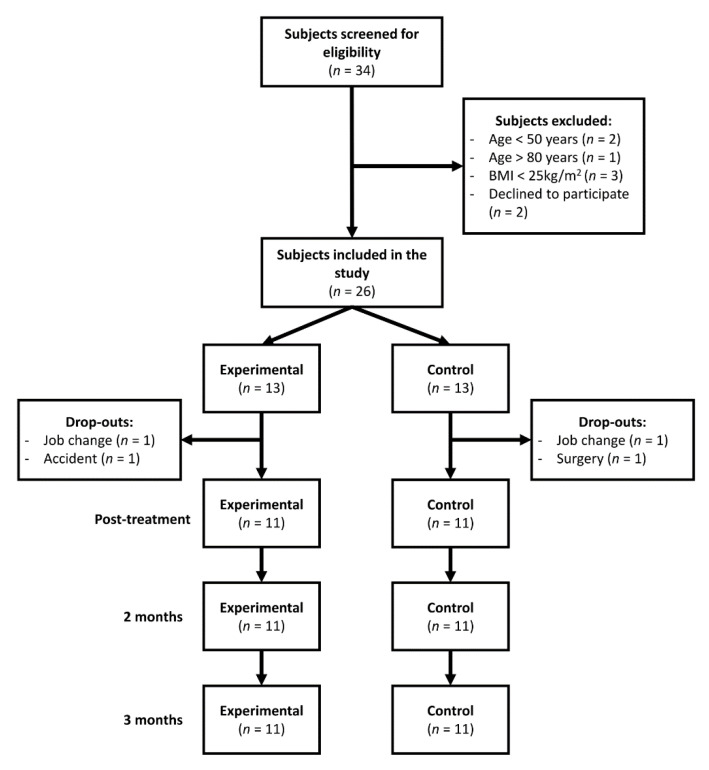
Flow diagram of subjects. Abbreviations: BMI, body mass index.

The study flow diagram is depicted in Figure 1. A total of 34 patients were screened for eligibility. Eight participants were excluded as they failed to fulfil the inclusion criteria. A total of 26 (15 women, 11 men) participants were finally included. They were randomized into two groups: experimental group (*n* = 13) and control group (*n* = 13). There were four dropouts during the study period, two in each group due to changing their city of residence. No adverse effects were reported in either group.

Baseline characteristics of the two intervention groups are presented in Table 1. There were no significant differences in baseline variables between groups (all *p* > 0.05).

### 3.1. Primary Outcomes: Self-Reported Knee Pain

Table 2 shows the values of VAS for each group in each moment in time. Statistically significant differences were found in the interaction of treatments and times (F_(3,60)_ = 5.807, *p* < 0.05; η_p_^2^ = 0.22). These differences were found in the experimental group between baseline (pre) and at three months (postmonth3) (mean difference, −31.81; 95% CI, −58.25 to −5.38; *p* = 0.013), and the effect size was considered large. Differences were found between the control and experimental groups, between the baseline measurement and the postmonth2 (*p* = 0.009) and postmonth3 (*p* = 0.008) measurements. Effect sizes were considered weak between the baseline and postmonth1 (d = 0.23) measurements; medium between baseline and postmonth2 (d = 0.46), and large between baseline and postmonth3 (d = 1.13) (Table 2). A clinically significant difference of 3.7 points in pain reduction at the end of treatment was found, in favor of the experimental group.

### 3.2. Secondary Outcome: Exercise Adherence 

The subjects in the experimental group showed significantly greater adherence to the exercise program compared to subjects in the control group at post36 follow-up (U = 23.50, *p* = 0.024) but not at post12 (U = 43.50, *p* = 0.43) and post24 (U = 39.00, *p* = 0.28) follow-ups. A description of adherence data is presented in Table 3.

### 3.3. Secondary Outcome: Knee Pain and Disability

Table 4 presents the data from the WOMAC questionnaires. No statistically significant differences were found in the WOMACT questionnaire within the groups between the baseline measurement (pre) and the rest of the post-measurements (F_(3,60)_ = 0.718, *p* = 0.521; η_p_^2^ = 0.03). No differences were found between both groups, between the baseline measurement and any of the measurements (*p* > 0.016). Effect sizes found were considered weak in all measurements (d = 0.23, d = 0.37, and d = 0.30).

Likewise, no significant differences were found in the WOMACD within either group between the baseline measurement (pre) and the rest of post-measurements (F_(3,60)_ = 0.718, *p* = 0.521; η_p_^2^ = 0.03). No differences were found between both groups, between the baseline and the rest of the measurements (*p* > 0.016). Effect sizes are considered weak in all measurements (d = 0.36, d = 0.38, and d = 0.17).

No differences were found in the WOMACR within the groups (F_(3,60)_ = 0.718, *p* = 0.521; η_p_^2^ = 0.03) or between groups (*p* > 0.016). The effect sizes found were weak in all measurements (d = 0.09, d = 0.11, and d = 0.10). No differences were found in the WOMACC within the groups (F(3,60) = 0.256, *p* = 0.857; η_p_^2^ = 0.01) or between groups (*p* > 0.016). The effect sizes found were weak in all measurements (d = 0.17, d = 0.39, and d = 0.35). None of the questionnaires analyzed presented clinically significant changes.

### 3.4. Secondary Outcome: Quality of Life

No significant differences were found within the groups in any of the questionnaires analyzed: SF12PCS (F(3,60) = 0.687, *p* = 0.564; η_p_^2^ = 0.03), SFP12MCS (F(3,60) = 1.150, *p* = 0.336; η_p_^2^ = 0.05), and SF12T (F(3,60) = 1.767, *p* = 0.163; η_p_^2^ = 0.08). Likewise, there were no statistically significant differences between the groups in any of the SF-12 questionnaires between the baseline measurement and the rest of the measurements (*p* < 0.016). Effect sizes were weak in all cases, except in SF12MCS, between the baseline measurement and postmonth2, when it was moderate (d = 0.54) and in SF12Total between the baseline measurement and at 12 months (d = 0.70) (Table 5).

## 4. Discussion

This study showed that adding aerobic exercises performed in an unloaded position to a conventional strengthening and stretching program resulted in superior effects in self-reported knee pain, knee pain and disability, and quality of life as compared to adding loaded aerobic exercises in overweight subjects with knee OA. Interestingly, beneficial effects in the experimental group were observed after administering 24 sessions of treatment and remained at the end of treatment, after 36 sessions. The percentage of adherence to treatment was higher in the experimental group (59%) as compared to that in the control group (41%). Given the inability some subjects with knee OA may have to perform aerobic training in loading positions due to pain and/or overweight, aerobic exercise in a sitting, unloaded position might constitute a safe alternative.

### 4.1. Self-Reported Knee Pain

Significant within group and between groups differences were observed in self-reported knee pain in favor of the experimental group between baseline measurement and measurements taken after 24 and 36 sessions of treatment. In addition, the reduction in self-reported knee pain in the experimental group at the end of treatment as compared to baseline was clinically meaningful (>20 mm) [21]. These results indicate that when adding aerobic exercises in a sitting, unloaded position to conventional exercises in overweight subjects with knee OA, at least 24 sessions may be necessary to expect a significant improvement in pain according to the VAS.

These results are in line with findings related to mechanical loading and the presence of pain in patients with knee OA [30,31]. In addition, Beckwée et al. [32] reported that the knee pain of subjects with signs of peak mechanical loading improved significantly after low-mechanical-load strength exercises but not after high-mechanical-load walking exercises [32]. Hence, the improved results of unloaded compared to loaded exercises, may be explained by a decreased mechanical knee load. On the contrary, the beneficial effects of exercises may also act through pathways other than mechanical ones [33,34]. Indeed, in light of recent insights into chronic musculoskeletal pain, it is suggested to consider knee OA pain from a multidimensional perspective with contributions of not only knee pathology (including joint load) but also psychological distress and pain neurophysiology [35]. For example, Wideman et al. reported that both pain catastrophizing and processes related to central sensitization predicted knee pain during a weight-bearing activity [31]. However, since these potential confounding factors have not been taken into account in the current study, further research is warranted. 

### 4.2. Knee Pain and Disability

The total WOMAC, WOMAC pain subscale, and WOMAC functional subscale scores only revealed a significant improvement in the experimental group when measured after 24 and 36 sessions of treatment. However, this improvement was not clinically meaningful for the experimental group when considering the differences observed in the total WOMAC score. These results are important as the pain and function of people with knee OA is significantly worse than that of the reference population [25]. Previous research showed beneficial effects for up to six months in the WOMAC pain subscale with a similar exercise program as used in the current study [36]. Our results, however, are not consistent with studies using walking as the aerobic exercise [37].

### 4.3. Quality of Life

Only the experimental group achieved significant improvements in quality of life measured with the SF-12 questionnaire. Our findings are in accordance with previous studies from Escalante et al. [38] and Beckwée et al. [39], in which exercise showed positive effects on quality of life, mental health, and depression in patients with knee OA. The slight drop in improvement in SF12MCS observed in the current study between post24 and post36 measurements (5.98 to 5.01 points) might be due to a lack of progression in exercise intensity. 

### 4.4. Adherence to Treatment

Although the researchers contacted the patients every 2 weeks, the adherence rate was low in the control group (58.05%) compared to that in the experimental group (86.10%) at post36 follow-up. This may be an important confounding factor since a dose–response relation exists for physical activity [40]. Patient adherence to exercise declines over time, so appropriate attention to this issue is advocated in people with knee OA, as reduced adherence may attenuate the benefits of exercise [41]. Furthermore, it seems that higher exercise adherence is associated with improved physical function in overweight and obese older adults with knee OA [22]. This indicates that barriers and facilitators to exercise should be identified and strategies to maximize long-term adherence, promoting adherence to exercise should be implemented [41].

### 4.5. Limitations

The main limitation of this study is the small sample size, which implies that the results of this study may not be generalized. In addition, the radiographic disease severity for each participant was unknown, so results may not be extrapolated to people with different degrees of knee OA. The absence of a control group receiving no-treatment raises the possibility that the positive results obtained in this study may not be due to the exercise program but due to other factors such as the natural history of the disorder or regression to the mean. However, due to the study design (randomization of the sample, baseline homogeneity of the study groups, and blinding of patients and external evaluators) and because both groups performed the same routine of strengthening and stretching exercises, it is unlikely that the results are due to other factors unrelated to the aerobic exercise in a sitting position.

Minimal clinically important difference was only established for some variables but not for others. Therefore, firm conclusions about the clinical relevance of findings related to the variables where no data existed could not be made. No progression in exercise intensity was done, which does not reflect common clinical practice.

## 5. Conclusions

An exercise program combining aerobic training in an unloaded position with strengthening and stretching exercises seems to produce superior beneficial effects in self-reported knee pain, knee pain and disability, and quality of life as compared to the same program but with aerobic training applied in a loaded position in overweight subjects with knee OA. However, more research is needed to draw definitive conclusions.

## Figures and Tables

**Table 1:** Baseline characteristics of the patients (*n* = 22).

Characteristic *	Control Group (*n* = 11)	Experimental Group (*n* = 11)	*p* Value
Sex (male/female)	6/5	4/7	0.670
Age (years)	60.27 (9.27)	68.55 (9.95)	0.057
Weight (kg)	76.61 (20.97)	79.96 (15.85)	0.677
Height (cm)	157.45 (10.62)	156.55 (10.73)	0.844
BMI (kg/m^2^)	30.64 (6.72)	32.31 (5.16)	0.464
VAS (0–100 mm)	43.18 (31.27)	63.90 (19.97)	0.079
WOMAC- total (0–96)	41.63 (22.58)	42.36 (15.51)	0.931
WOMAC-pain subscale (0–20)	8.90 (5.20)	8.63 (2.87)	0.881
WOMAC-stiffness subscale (0–8)	4.09 (2.50)	3.09 (2.87)	0.395
WOMAC-function subscale (0–68)	28.63 (15.85)	30.63 (11.44)	0.738
SF12PCS (0–100)	32.98 (8.37)	35.79 (8.78)	0.202
SF12MCS (0–100)	48.70 (9.29)	47.86 (11.62)	0.854

* Values are presented as mean (SD). Abbreviations: BMI, body mass index; WOMAC, Western Ontario and McMaster Universities Osteoarthritis Index; SF12MCS, Short Form 12 Health Survey—Mental Component Summary; SF12PCS, Short Form 12 Health Survey—Physical Component Summary; VAS, Visual Analogue Scale.

**Table 2:** Self-reported knee pain data.

Group	Baseline	Post12	Post24	Post36
VAS * (0–100 mm)				
Control Group	43.18 (31.27)	46.72 (30.56)	54.81 (28.49)	48.82 (32.41)
Experimental Group	63.90 (19.97)	53.90 (36.50)	38.27 (32.25)	32.09 (26.78)
**Within-group change score from baseline †**				
Control Group		3.54 (−17.77, 24.86)	11.63 (−13.37, 36.65)	5.64 (−18.73, 30.02)
Experimental Group		−10.00 (−24.90, 4.90)	−25.63 (−39.38, −11.89) *~*	−31.81 (−58.25, −5.38) *~*
Between-group difference in change score ‡		−13.54 (−37.90, 10.81); d = 0.23	−37,27 (−63.99, −10.55) *~*; d = 0.46	−37.46 (−64.10, −10.82) *~*; d = 1.13

* Values are mean (SD). † Compared to pre-treatment. ‡ Values mean difference (95% confidence interval). ~ Statistically significant differences (*p* < 0.05). d = 2t/√g. Abbreviations: VAS, Visual Analogue Scale. Post12, post 12 treatment sessions, Post24, post 24 treatment sessions, Post36, post 36 treatment sessions.

**Table 3:** Exercise adherence.

Group	Post12	Post24	Post36
Control Group *	95.83% (75%–100%)	72.92% (50%–95.83%)	58.05% (38.89%–77.78%)
Experimental Group *	100% (91.65%–100%)	91.65% (87.47%–97.92%)	86.10% (77.76%–94%)

* Values are median (1st–3rd quartiles). Post12, post 12 treatment sessions, Post24, post 24 treatment sessions, Post36, post 36 treatment sessions.

**Table 4:** WOMAC questionnaire data.

Group	Baseline	Post12	Post24	Post36
**WOMAC-total**				
Control group	41.63 (22.58)	44.00 (21.67)	39.54 (21.18)	34.27 (22.95)
Experimental Group	42.36 (15.51)	36.18 (18.29)	35.16 (17.66)	30.63 (16.13)
**Within-group change score from baseline †**				
Control Group		2.36 (−10.96, 15.69)	−2.09 (−13.60, 15.69)	7.36 (−18.02, 15.69)
Experimental Group		−6.18 (−14.23, 1.86)	−7.18 (−13.18, −1.17) *~*	−11.72 (−20.41, −3.03) *~*
Between-group difference in change score ‡		−8.54 (−23.12, 6.03); d = 0.23	−5.09 (−17.25, 7.06); d = 0.37	−4.36 (−17.23, 8.50); d = 0.30
WOMAC–pain subscale *				
Control Group	8.90 (5.20)	8.45 (4.94)	7.63 (3.85)	6.45 (4.45)
Experimental Group	8.63 (2.87)	6.81 (3.54)	6.00 (3.60)	5.50 (3.17)
**Within-group change score from baseline †**				
Control Group		−0.36 (−3.34, 2.61)	−1.27 (−3.78, 1.24)	−2.45 (−5.06, −0.15)
Experimental Group		−1.81 (−3.85, 0.21)	−2.63 (−4.80, −0.46) *~*	−3.18 (−6.21, −0.15) *~*
Between-group difference in change score‡		−1.45 (−4.83, 1.92); d = 0.36	−1.36 (−4.47, 1.74); d = 0.38	−0.72 (−4.45, 3.02); d = 0.17
WOMAC-stiffness subscale *				
Control Group	4.09 (2.50)	3.37 (1.95)	3.36 (2.01)	3.27 (2.00)
Experimental Group	3.09 (2.87)	2.19 (2.01)	2.54 (1.91)	2.09 (1.75)
**Within-group change score from baseline †**				
Control Group		−0.72 (−2.34, 0.89)	−0.74 (−2.17, 0.71)	−0.81 (−2.22, 0.58)
Experimental Group		−0.90 (−1.78, −0.03)	−0.54 (−1.83, 0.74)	−1.00 (−2.12, 0.12)
Between-group difference in change score ‡		−0.18 (−1.90, 1.59); d = 0.09	0.20 (−1.62, 1.99); d = 0.11	−0.19 (−1.86, 1.50); d = 0.10
WOMAC–function subscale *				
Control Group	28.63 (15.85)	32.18 (15.37)	28.54 (15.98)	24.90 (17.47)
Experimental Group	30.63 (11.44)	28.00 (13.89)	26.63 (13.15)	23.09 (12.10)
**Within-group change score from baseline †**				
Control Group		3.55 (−4.48, 10.57)	−0.09 (−8.47, 8.29)	−3.72 (−11.75, 4.29)
Experimental Group		−2.64 (−9.66, 4.39)	4.00 (−0.38, 8.38)	−7.54 (−13.46, −1.62) *~*
Between-group difference in change score ‡		3.19 (−12.65, 19.04); d = 0.17	−3.90 (−12.77, 4.95); d = 0.39	−3.81 (−13.15, 5.51); d = 0.35

* Values are mean ± SD. † Compared to pre-treatment. ‡ Values mean difference (95% confidence interval). ~ Statistically significant differences (*p* < 0.05). d = 2t/√g. Abbreviations: WOMAC, Western Ontario and McMaster Universities Osteoarthritis Index. Post12, post 12 treatment sessions, Post24, post 24 treatment sessions, Post36, post 36 treatment sessions.

**Table 5:** SF12 questionnaire data.

Outcome	Baseline	Post12	Post24	Post36
SF12PCS *				
Control group	32.98 (8.37)	35.79 (8.78)	36.68 (9.20)	36.80 (10.89)
Experimental Group	38.29 (10.40)	40.98 (6.38)	37.73 (8.08)	37.29 (10.20)
**Within-group change score from baseline †**				
Control Group		2.80 (−5.11, 10.73)	3.70 (−3.66, 11.06)	3.81 (−4.00, 11.64)
Experimental Group		2.69 (−4.19, 9.57)	−0.56 (−7.34, 6.23)	−1.00 (−10.12, 8.12)
**Between-group difference in change score ‡**		−0.11 (−9.94, 9.71); d = 0.00	−4.25 (−13.85, 5.34); d = 0.29	−4.81 (−16.07, 6.43); d = 0.22
SF12MCS *				
Control Group	48.70 (9.29)	48.12 (9.80)	47.90 (9.41)	48.73 (9.48)
Experimental Group	47.86 (11.62)	50.13 (11.24)	53.05 (9.97)	53.62 (9.36)
**Within-group change score from baseline †**				
Control Group		−0.48 (−5.27, 4.31)	−0.79 (−6.74, 5.16)	0.03 (−8.36, 8.43)
Experimental Group		2.27 (−3.30, 7.85)	5.19 (0.41, 5.16) ~	−5.76 (−1.60, 13.12)
**Between-group difference in change score ‡**		2.75 (−4.13, 9.64); d = 0.23	5.98 (−1.16, 13.12); d = 0.54	5.01 (−4.73, 16.18); d = 0.48

* Values are mean ± SD. † Compared to pre-treatment. ‡ Values mean difference (95% confidence interval). ~ Statistically significant differences (*p* < 0.05). d = 2t/√g. Abbreviations: SF12MCS, Short Form 12 Health Survey—Mental Component Summary; SF12PCS, Short Form 12 Health Survey—Physical Component Summary.

## Supplementary Materials

The following are available online at https://www.mdpi.com/2077-0383/9/12/4113/s1, Supplementary Material S1: Warming-up and cooling-down exercises included in the aerobic training for both the experimental and control group, Supplementary Material S2: Aerobic exercises, Supplementary Material S3: Strengthening exercises, Supplementary Material S4: Stretching exercises.
