# Supplementary material for: Effectiveness of a Program Combining Strengthening, Stretching, and Aerobic Training Exercises in a Standing versus a Sitting Position in Overweight Subjects with Knee Osteoarthritis: A Randomized Controlled Trial
Source: J Clin Med. 2020 Dec 20;9(12):4113. doi: 10.3390/jcm9124113 (PMC7766867; doi:10.3390/jcm9124113)
Supplement: Supplementary file 1 [file jcm-09-04113-s001.pdf]

Supplementary material 1. Warming-up and cooling-down exercises included in the aerobic training for both the experimental and control group.

**<u>Sitting</u>**	
**Starting position for all exercises:** seated with 90°knee flexion, feet flat on the floor and leaning back on the chair.	
**Execution**: With hands on thighs lift feet a few centimetres off the ground alternately for 1 minute.	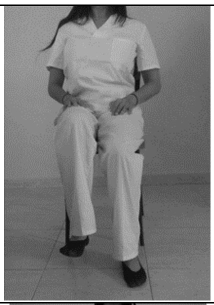
**Execution**: Lift feet a few centimeters off the ground alternately for 1 minute while performing posterior shoulder circumduction.	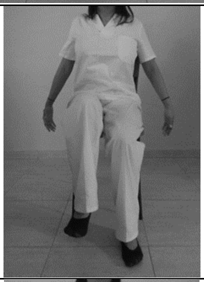
**Execution**: Lift feet a few centimeters off the ground alternately for 1 minute while raising shoulders alternately.	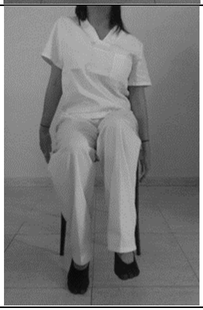
**<u>Standing</u>**	
**Starting position for all exercises**: standing	
**Execution**: gentle walking	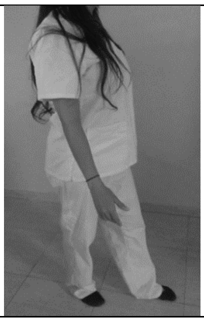

Supplementary material 2. Aerobic exercises.

**Sitting**	
**Starting position for all exercises:** seated with 90°knee flexion, feet flat on the floor and leaning back on the chair.	
**Execution**: Perform knee extension and shoulder flexion with alternate contralateral elbow extension.	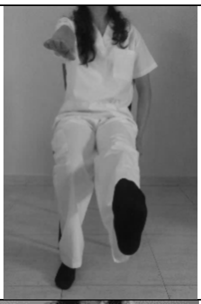
**Execution**: Touch contralateral knee alternately and vigorous walking movement	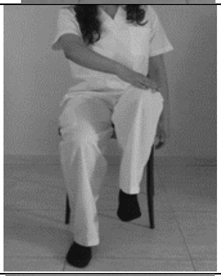
**Execution**: Hip abduction and clap hands above head alternately.	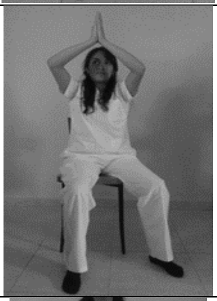
**Execution**: brisk walking movement alternately flexing elbows.	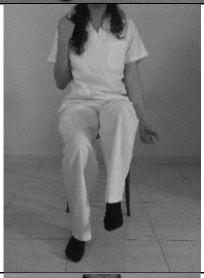
**Execution**: brisk walking movement with hands on contralateral shoulders simultaneously in front of trunk.	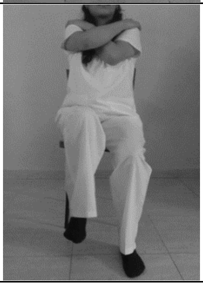
**Standing**	
**Starting position for all exercises**: standing<br>**Execution**: Brisk walking as tolerated by patient	

Supplementary material 3. Strengthening exercises

**<u>Quadriceps</u>**<br>**Patient's position**: Supine with a small ball below the popliteal space.<br>**Execution**: Push with the back of the knee downwards trying to squash the ball contracting the quadriceps. 10 repetitions are performed on each leg with 10 seconds contraction and 2 seconds rest for all exercises of this figure.	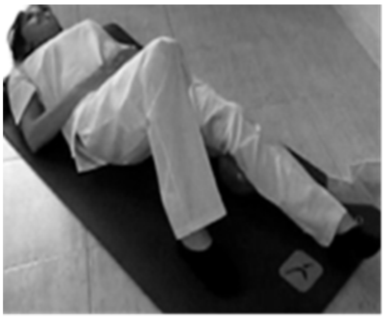
**<u>Hamstrings</u>**<br>**Patient's position:** Prone with knee flexed at 90 °.<br>**Execution**: Place a ball in the popliteal space of both knees and bring to flexion exerting pressure on the ball; when relaxing do not go to full extension.	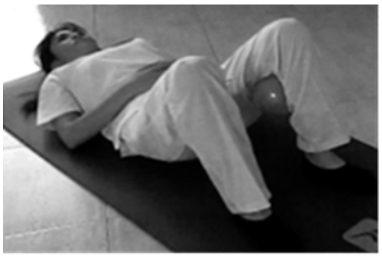
**<u>Triceps Surae</u>**<br>**Patient's position**: Decubitus supine.<br>**Execution**: Perform bilateral ankle plantar flexion using an elastic band for resistance	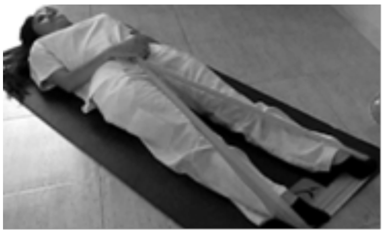
**<u>Hip abductors</u>**<br>**Patient's position**: Decubitus supine with 45° hip flexion and 90° knee flexion.<br>**Execution**: Place knees apart attempting to overcome resistance from the elastic band.	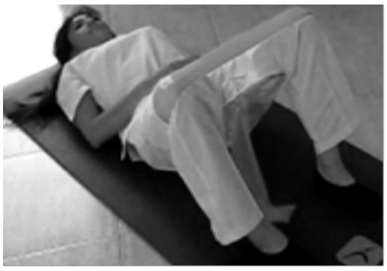
**<u>Hip adductors</u>**<br>**Patient's position**: Decubitus supine with 45° hip flexion and 90° knee flexion.<br>**Execution**: Bring knees together squashing the ball placed between the knees.	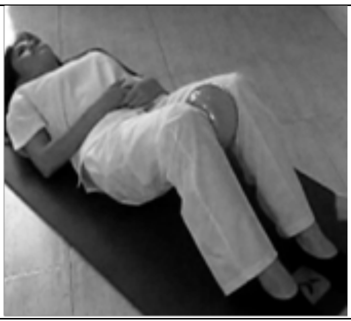

Supplementary material 4. Stretching exercises

**Quadriceps**<br>**Patient's position**: lateral decubitus.<br>**Execution**: Hold the ankle of the leg to stretch and take the heel towards the ipsilateral gluteus.<br>10 repetitions are performed on each leg with 10 seconds stretching and 2 seconds rest for all exercises of this figure.	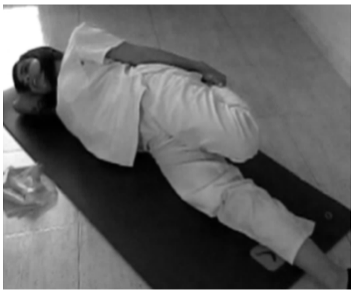
**Hamstrings and Triceps Surae**<br>**Patient's position**: Supine position with hip flexion and knee extension to tolerance<br>**Execution**: Using the elastic band, bring the ankle in dorsiflexion until feeling a slight tension.	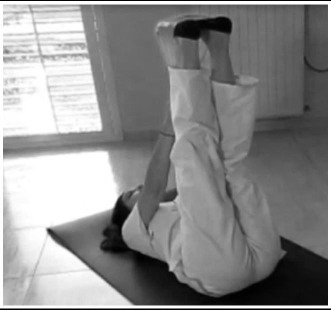
**Hip abductors**<br>**Patient's position:** Seated with legs stretched.<br>One leg is placed over the other with a knee flexed to patient's tolerance<br>**Execution:** Take the top leg adducted across to the furthest extent to the midline. The movement can be reinforced using a hand in that direction.	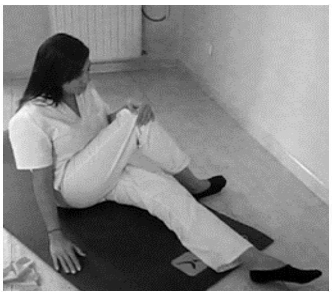
**Hip adductors**<br>**Patient's position:** Decubitus position with 45° hip flexion, knee at 90 °and feet together.<br>**Execution:** Separate legs to patient tolerance. Place hands on knee mid side to force the movement in that direction.	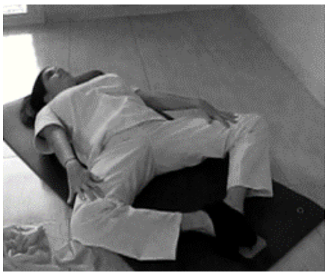
